# Reproductive concerns among adolescent and young adult cancer survivors: A scoping review of current research situations

**DOI:** 10.1002/cam4.4708

**Published:** 2022-03-24

**Authors:** Jianfei Xie, Qian Sun, Yinglong Duan, Qinqin Cheng, Xiaofei Luo, Yi Zhou, Xiangyu Liu, Panpan Xiao, Andy S. K. Cheng

**Affiliations:** ^1^ Department of Rehabilitation Third Xiangya Hospital of Central South University Changsha China; ^2^ School of Nursing Sun Yat‐Sen University Guangzhou China; ^3^ Department of Emergency Third Xiangya Hospital of Central South University Changsha China; ^4^ Nethersole School of Nursing Chinese University of Hong Kong Hong Kong China; ^5^ Xiangya Nursing School Central South University Changsha China; ^6^ Health Management Center of Hunan Cancer Hospital Changsha China; ^7^ Department of Rehabilitation Hong Kong Polytechnic University Kowloon China

**Keywords:** adolescent, cancer, reproductive concerns, survivorship, young adult

## Abstract

Fertility is a significant concern among adolescent and young adult (AYA) cancer survivors and their caregivers, especially after their completion of cancer treatment programs. Concerns about fertility affect not only cancer patients' psychological well‐being, but also all aspects of their medical treatments, including treatment protocol, decision‐making, and treatment adherence. In this scoping review, the PubMed, CINAHL, Web of Science, Embase, CNKI, and Wanfang electronic databases were searched according to the guidelines of the Preferred Reporting Items for Systematic Reviews and Meta‐Analysis Extension for Scoping Reviews. The searches identified 669 articles, 54 of which met the inclusion criteria. Reviewers extracted the data on the study characteristics, measurements, positive factors, negative factors, and additional themes. This scoping review included studies from 10 countries. Most studies were quantitative using a cross‐sectional design. The prevalence of reproductive concerns among AYA cancer survivors ranged from 44% to 86%, and 28% to 44% of the survivors experienced moderate to severe concerns. The specific implementation of fertility consultation, including timing, consult frequency, and content, deserves ongoing exploration.

## Graphical abstract

Current research situations about reproductive concerns in adolescent and young adult cancer survivors.

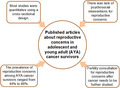

**Lay Summary**

This scoping review overviewed the current research situations about reproductive concerns among adolescent and young adult (AYA) cancer survivors and showed the prevalence of reproductive concerns among AYA cancer survivors ranged from 44% to 86%, and 28% to 44% of the survivors experienced moderate to severe concerns. There was few research focusing on interventions to alleviate reproductive concerns. Therefore, theoretical frameworks for this problem should be explored, and appropriate psychotherapy should be designed to alleviate their concerns.

**Precis for use in the Table of Contents**

The prevalence of reproductive concerns among AYA cancer survivors ranged from 44% to 86%, and 28% to 44% of the survivors experienced moderate to severe concerns. There lacks an appropriate interventions to alleviate reproductive concerns among AYA cancer survivors.

## 1 INTRODUCTION

Fertility plays an important role in the continuation of human life, and young adulthood is the prime time to have own biological children. However, among cancer survivors, reproductive function is often impaired or interrupted due to the destructive nature of the cancer itself or due to the reproductive toxicity of the cancer treatment. Thus, there are concerns about the fertility potential of patients diagnosed with cancer, especially among adolescent and young adult (AYA) (aged 15–39 years) cancer survivors.^1^ In 2018, the American Society of Clinical Oncology (ASCO) issued updated evidence‐based clinical practice guidelines on fertility preservation pointing out that healthcare providers caring for pediatric and adult cancer patients should address the possibility of infertility with patients and/or their parents during their reproductive years; the ASCO guidelines also advised that practitioners should be prepared to discuss fertility preservation options and/or to refer all potential patients to appropriate reproductive specialists as early as possible before initiation of treatment.^2^ However, although the proportion of fertility counseling initiated by oncologists has increased, less than half of cancer patients are satisfied with the fertility counseling they receive, and referrals to fertility specialists remain low.^3^, ^4^ Moreover, some cancer patients have difficulty making decision about fertility preservation partly for their lack of adequate information on fertility preservation (lack of information, the timing of the information, and patient‐provider communication) and lack of referrals from oncology centers to reproductive clinics.^5^ One cross‐sectional study in Canada showed that less than 10% of AYA cancer patients received fertility preservation services.^6^

With the steadily increasing 5‐year survival rate of cancer patients,^7^ more than 85% of AYA cancer survivors have a strong desire for a child and be biological parenthood for themselves,^8^ and fertility has become a significant concern for patients and/or their family and caregivers after the completion of cancer treatment^9^, ^10^, ^11^, ^12^. Gorman et al.^13^, ^14^ posited six core domains of patients' reproductive concerns during cancer survivorship. These domains include 1) fertility potential, 2) disclosure to partner of fertility status, 3) the child health, 4) personal health, 5) acceptance, and 6) achieving pregnancy/becoming pregnant. A recent systematic review showed that fertility concerns can last as long as decades after a cancer diagnosis and constitute an important source of AYA cancer survivors' psychological distress.^15^ Over time, this can easily lead to the development of psychological disorders and increase the financial burden on patients. In addition to psychological effects, reproductive concerns also affect all aspects of medical treatment, including treatment protocol decision‐making and adherence.^16^, ^17^ Ultimately, all these factors contribute to the poor long‐term quality of life among AYA cancer survivors.^18^

To provide directions for improving the quality of life, it is significant to learn about the level, influencing factors, and clinical interventions of reproductive concerns in AYA cancer survivorship. The objective of this scoping review was to evaluate the literature on reproductive concerns among AYA cancer survivors after the completion of cancer treatment, to identify research gaps in the current literature and to describe future research directions to alleviate reproductive concerns.

## 2 METHOD

We performed our scoping review guided by the six steps illustrated by Arskey and O′Malley^19^: (1) identify the research questions, (2) identify relevant studies, (3) select studies, (4) chart the data, (5) collate, summarize, and report the results of the selected studies, and (6) consult with stakeholders. Our scope review captured the diversity of research and enabled researchers to review studies that explored reproductive concerns using different methodologies, identify research gaps in the literature and guide future research directions. We used the PRISMA reporting guidelines for the full report. This was a secondary data analysis which was based on published aggregate data. Neither informed consent to participate nor ethical approval is required.

### 2.1 Research Questions

To accomplish the aims of the study, the following research questions were identified:

1. How have reproductive concerns among AYA cancer survivors been assessed?
2. What have assessment on reproductive concerns among AYA cancer survivors shown?
3. What factors may associate with the level of reproductive concerns among AYA cancer survivors?
4. Are there interventions to alleviate reproductive concerns among AYA cancer survivors?

### 2.2 Information Sources

We conducted a comprehensive search of the PubMed, CINAHL, Web of Science, Embase, CNKI, and Wanfang electronic databases in August 2021. We searched the above databases using a combination of the following keywords: child, OR young adults, OR adolescents, OR childbearing age, AND cancer survivorship, OR childhood cancer survivors, OR cancer, OR malignancy, AND fertility concern*, OR infertility concern*, OR reproductive concern*, OR fertility worries, OR fertility worry.

### 2.3 Inclusion and Exclusion Criteria

Inclusive criteria were met if studies (1) were published in peer‐reviewed journals in English or Chinese; (2) applied quantitative, qualitative, or mixed methods; (3) answered the review question; and (4) included study sample groups composed at least 50% by participants aged 15–39 years old who had completed cancer‐related treatment. Exclusion criteria were met if studies (1) were focused mainly on fertility, fertility preservation, or fertility‐related (psychological) distress; (2) reported overall outcomes of AYAs and older adults, such as the results of AYAs that could not be extracted and described; (3) included study sample groups composed over 50% by survivors older than 39 years; or (4) were case studies, review articles, correspondence, gray literature, or conference abstracts.

### 2.4 Study selection

After removing duplicates, titles were filtered using the conservative method described by Higgins and Green^20^ (Sections 7.2.3 and 7.2.4 of 2011), and studies that were not clearly relevant to the study question were removed by the first author. Two investigators then applied the inclusion criteria to the remaining titles and abstracts to ensure that pertinent studies were included. The impact or investigation of reproductive concern had to have been reported in the Abstract to ensure that it was the primary topic of the study. Inclusion criteria and screening results were compared, and any discriminations were resolved through discussion and review of the full text until a consensus was reached.

### 2.5 Data extraction

Two researchers (Sun Q and Xiao P) extracted data from eligible studies using electronic forms to ensure accuracy. The following information was extracted: study authors, publication year, nationality of the author, study design, type of cancer and age, measurements, and main outcomes. The results were depicted with statistics and thematic analysis. Although the PRISMA: Extension for Scoping Reviews does not ask for bias analysis across studies, we acknowledge the risk of selectively reporting themes, particularly in qualitative studies.

## 3 RESULTS

**FIGURE 1:**
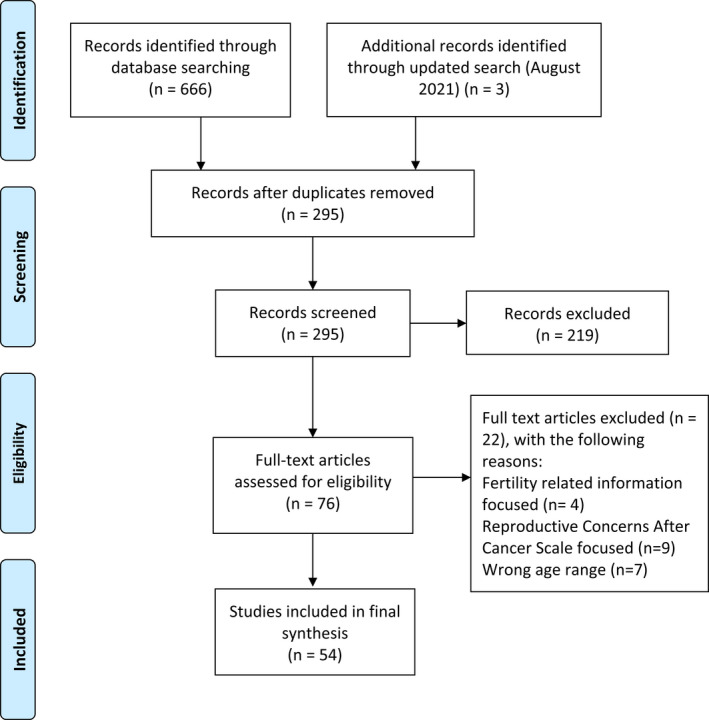
Flow diagram of identifying literature

Database searching yielded 669 articles, from which 374 repeated articles were removed. In the remaining 295 articles, 219 articles were excluded after screening the abstract and title. After screening the full text of 76 articles, 22 articles did not satisfy the inclusion criteria and were excluded for the following reasons: focus on fertility‐related information (*n* = 4); focus on reproductive concerns after cancer scale (*n* = 9); focus on wrong age range (*n* = 7); French language (*n* = 1); and inability to access the full text (*n* = 1). The flowchart of the study selection process is shown in Figure 1. The majority of studies were quantitative and applied a cross‐sectional (*n* = 33) or observational cohort (*n* = 4) design. Only one study used a mixed method design, and four studies reported experimental data.

### 3.1 Study characteristics

As shown in Table S1, the publication year of the eligible articles in this scoping review ranged from 1999 to 2021. Among these articles, 33.3% were conducted in the USA (*n* = 18), 33.3% in China (*n* = 18), and 5.6% in the United Kingdom (*n* = 3). In this review, AYA‐aged survivors included childhood and AYA cancer survivors; 37 studies included only survivors who were diagnosed in adolescence and/or young adulthood (i.e., aged 15–39 years), while eight studies included participants diagnosed across childhood and young adulthood (e.g., aged 14–18 years), and one study focused on survivors diagnosed in patients 15 years of age or younger. Forty studies included only females, three included only males, and 11 included both male and female cancer survivors. Most studies focused on survivors of various cancers (*n* = 19) or on breast cancer survivors (*n* = 20). Three themes emerged as important concepts related to reproductive concerns: (1) reproductive concerns, encompassing the prevalence of reproductive concerns, and other aspects of reproductive concerns; (2) influencing factors (e.g., depression, fertility consultation); and (3) interventions for reproductive concerns.

### 3.2 Reproductive concerns

Various measures were used to examine reproductive concerns, which were reported as a primary outcome in 40 articles (Supplement File). Measurements of reproductive concerns involved the Reproductive Concerns Scale (RCS),^21^, ^22^, ^23^ the Reproductive Concerns After Cancer Scale (RCAC),^18^, ^24^, ^25^, ^26^, ^27^, ^28^, ^29^, ^30^, ^31^, ^32^, ^33^, ^34^, ^35^, ^36^, ^37^, ^38^, ^39^, ^40^, ^41^, ^42^, ^43^, ^44^, ^45^, ^46^, ^47^, ^48^ the Female Sexual Function Inventory (FSFI),^49^ the Fertility Problem Inventory,^50^ the Cancer‐specific version of the Leipzig Questionnaire of Motives to have a Child (LQM‐C),^51^ the Fertility Issues Survey,^52^, ^53^, ^54^ and the Fertility Issues and Outcomes Scale.^55^ Hammond, Abrams, and Syrjala used a digital scoring method (scored from 0 = not at all to 10 = very much) to measure the level of concern about infertility.^56^ Five studies used a self‐designed scale to examine reproductive concerns.^8^, ^57^, ^58^, ^59^, ^60^ Reproductive concerns were also discussed by survivors in qualitative studies.^9^, ^27^, ^43^, ^54^, ^61^, ^62^, ^63^, ^64^, ^65^, ^66^, ^67^, ^68^

### 3.3 Prevalence of reproductive concerns

Due to the diversity of measurement tools used in the different studies, it is hard to determine the overall prevalence of AYA cancer survivors with reproductive concerns. In studies using the RCAC, 58% to 61% of AYA cancer survivors reported high concerns on at least one dimension, and 28% to 44% of the survivors reported moderate to high overall reproductive concerns, with the total scores for reproductive concerns ranging from 56.45 ± 8.18 to 65.73 ± 12.36.^29^, ^31^, ^34^, ^36^, ^37^, ^38^, ^41^, ^44^, ^45^, ^47^ Compared with healthy controls, breast cancer survivors presented higher potential fertility concerns.^24^ As assessed using the RCS, high reproductive concerns were reported by 56% of breast cancer survivors^30,^ and no group differences in RCS mean scores were found between cancer survivors and infertile women without cancer.^21^ As assessed using the Fertility Issues Survey, 44% to 80% of survivors expressed certain level of concern regarding fertility, and survivors expressed greater concerns about fertility than their age‐ and gravidity‐matched controls.^52^, ^53^, ^69^ In a study using the Fertility Issues and Outcomes Scale, 51% were concerned about becoming infertile after treatment.^55^ Korte et al.^8^ and Schover et al.^60^ used a self‐designed scale to measure reproductive concerns and reported that both patients (86.1%) and their parents (96.3%) expressed a fierce desire for their own (or for their child's) biological reproduction, and 76% of those who were currently childless hoped to have children in the future. Partridge et al.^58^ found that 57% of young survivors with breast cancer recalled having had infertility concerns at diagnosis. Throughout the studies, female AYA cancer survivors reported more reproductive concerns than male survivors.^36^, ^37^ According to the qualitative data of cancer survivors, sexual dysfunction was identified as a substantial problem.^9^, ^62^, ^64^

### 3.4 Other aspects of reproductive concerns

Additional concepts about reproductive concerns were also reported in these studies. Three themes about reproductive concerns were extracted: worry and remorse, desire for communication and support, and demand for reproductive knowledge.^54^ Worry and remorse included partnership concerns and fertility concerns. Partnership concerns included fear of having to receive treatment without the support of a partner, fear of being rejected by a potential partner, and fear of wasting valuable time in diagnosis and treatment. Fertility concerns included the difficulty of having children and the choice to pursue‐assisted reproductive technology.^62^ Hammond, Abrams, and Syrjala^56^ reported that 25% of survivors who underwent myeloablative stem cell transplant expressed moderate to high levels of infertility concerns compared with 7% of the controls, and survivors who had no children before stem cell transplantation were at greater risk for fertility concerns 10 years later. Meanwhile, 65% of survivors reported that they were worried that the risk of hereditary cancer would be passed on to their children.^40^ Moreover, fertility concerns changed over time. Many women with cervical cancer who had undergone radical trachelectomy had reproductive problems for 6 months. Concerns about pregnancy seemed to decline after surgery; however, concerns about pregnancy seemed to increase after tracheotomy.^49^

### 3.5 Influencing factors

Factors associated with fertility concerns were identified in these studies and can be divided into three categories. As shown in Table 1, first, demographic factors included marital status, educational level, religious belief, and the number of children. Married women reported fewer psychosocial problems than single women,^53^ and survivors with lower education levels, no religious beliefs, and a lower number of children had higher levels of reproductive concerns.^28^, ^31^, ^34^, ^35^, ^38^ Moreover, cancer survivors whose primary caregiver was a sibling reported higher reproductive concern scores^35^. Second, clinical enablers included higher vigilance regarding reproduction‐related cues, frequent chemotherapy, acceptance of radioactive I^131^ therapy, being nulliparous at diagnosis, and reporting treatment‐related ovarian damage.^22^, ^26^, ^31^, ^34^, ^41^, ^44^ Compared to breast cancer patients, thyroid cancer patients had a lower level of reproductive concerns.^38^ Finally, psychosocial enablers included negative body image, great decisional conflict, poor social relational quality, and low self‐disclosure scores.^27^, ^28^, ^37^, ^47^ Young cancer survivors with a high risk of fertility impairment, an unfulfilled desire to have children, attachment anxiety, and depression had a higher level of reproductive concerns.^26^, ^31^, ^34^, ^35^, ^38^, ^44^ Young adult cancer survivors who received fertility counseling were more likely to have high levels of reproductive problems than those who do not receive fertility counseling.^29^, ^45^ However, if survivors received in‐depth reproductive counseling prior to treatment, they had lower levels of reproductive concerns.^23^ In addition, reproductive concerns fully mediated the relationship between the importance of parenting and quality of life, and was reported to have association with depression.^24^, ^25^, ^30^, ^39^

**TABLE 1:** Identified positive and negative influencing factors of reproductive concerns in adolescent and young adult cancer survivors

	Positive influencing factors	Negative influencing factors
Demographic factors	The high number of children^28^, ^31^, ^34^, ^35^, ^38^, ^41^, ^44^ Unmarried^35^, ^53^ High education level^34^, ^38^, ^44^ The primary caregiver is their child^35^	The low number of children^28^, ^31^, ^34^, ^35^, ^38^, ^41^, ^44^ Married^35^, ^53^ No religion^31^ Low education level^34^, ^38^, ^44^ The primary caregiver is sibling^35^ Prior difficulty conceiving^58^
Clinical factors	Low vigilance regarding reproduction‐related cues^26^ Small number of chemotherapy^31^ Be diagnosed as thyroid cancer^38^ Fertility preservation^29^, ^37^ Squamous cell carcinoma^41^	High vigilance regarding reproduction‐related cues^26^ large number of chemotherapy^31^ Accepted radioactive I^131^ therapy^44^ Be diagnosed as breast cancer^38^ Adenocarcinoma and adenosquamous cell carcinoma^41^ Being nulliparous at diagnosis^53^ Reporting treatment‐related ovarian damage^22^
Psychosocial factors	Less decisional conflict^27^ Well social relational quality^28^ Well family function^34^, ^46^ Low fertility intention^31^, ^46^ Low attachment anxiety^31^ Low degree of depression^24^, ^25^, ^30^, ^39^, ^44^ High self‐disclosure score^35^ Positive body image^37^ In‐depth reproductive health counseling^23^ Well psychosocial adaptation^27^	The desire to have a (or another) biological child^38^, ^41^ Greater decisional conflict^27^ Poor social relational quality^28^ Poor family function^34^, ^46^ High fertility intention^31^, ^46^ High attachment anxiety^31^ High degree of depression^24^, ^25^, ^30^, ^39^, ^44^ Low self‐disclosure score^35^ Negative body image^37^ Fertility counseling^29^, ^45^ Poor psychosocial adaptation^27^

### 3.6 Interventions for reproductive concerns

Wang et al.^70^ explored the effects of fertility consultation and education intervention and found that following consultation and education, the level of cancer‐related fertility knowledge was improved and reproductive concerns were alleviated among young gynecological malignant cancer patients. Previous studies also found that mindfulness‐based stress reduction, mindfulness training, and intimacy enhancement therapy could effectively relieve reproductive concerns and fear of cancer recurrence for young female cancer patients.^32^, ^42^, ^48^

## 4 DISCUSSION

We sought to explore the available literature on reproductive concerns in AYA cancer survivors who ranked fertility among their top three life goals. This scoping review found that reproductive concerns universally occurred in areas of China, America, and some European nations, and concerns related psychological interventions were scarce. However, for some economically less developed regions, such as India and areas of Africa, which have high cancer rates and high reproductive rates,^7^, ^71^ few studies have been published to date on reproductive concerns in patients with cancer, particularly those of AYA cancer patients. This may be a research gap, and scholars should further explore the prevalence and level of reproductive concerns in those areas and consider how concerns vary in different cultural and economic contexts. In addition, many cross‐sectional and longitudinal studies were conducted in small regional areas and with limited numbers of patients. Large sample studies with long‐term follow‐up are needed for definitive studies in the future.

Our findings showed that the prevalence of reproductive concerns in AYA cancer survivors ranged from 44% to 86% and that 28% to 44% of survivors experienced moderate to severe concerns. Although cancer patients do have a risk for impaired fertility, Bartolo et al.^24^ suggested that young females with breast cancers have identical concerns about fertility potential but a lower quality of life than infertile women, which indicates that cancer survivors have greater difficulty in adjusting (or experience a “double trauma” effect) disease and potential fertility damage. Lack of adequate information about reproductive options seemed to have a relationship with negative mood and increased distress in cancer survivors. However, two cross‐sectional studies showed that fertility counseling before treatment linked with greater reproductive concerns. This suggests that the quality of fertility counseling is not high enough to alleviate concerns.^29^, ^45^ In addition, consultation, to some extent, makes patients more aware of their actual infertility risk. Hence, the specific implementation of fertility consultation, including timing, consultant, frequency, and content, deserves ongoing exploration.

Child health, personal health, and fertility potential were the top three of the six dimensions of fertility concerns identified by AYA cancer patients in both quantitative and qualitative studies. In comparison, dimensions such as acceptance and achieving pregnancy/becoming pregnant received less attention from both male and female patients. The results of this research suggest that AYA cancer patients have a high perception of the risk of infertility and overemphasize the health problems that childbirth after cancer may bring to themselves and their children. In contrast, they tend to ignore their own emotional responses to infertility risks and appropriate remedies. These two aspects keep patients trapped in a vicious cycle of constant negative emotions and coping behaviors that ultimately make their concerns circular and compounding. In fact, reproductive concerns, just as fears of cancer recurrence, are, in essence, a normal concern that arises after cancer diagnosis and treatment, with high levels of concern associated with impaired quality of life.^72^ Currently, the theme of fear of recurrence after cancer (in theory and in practice) has been well studied, encompassing psychological cognitive processing, theoretical models, psychological interventions, etc.^73^, ^74^; however, little is known about these aspects as they manifest in fertility concerns among AYA cancer patients. Therefore, studies on reproductive concerns can draw on existing work conducted on the fear of cancer recurrence for guidance both to better understand the internal psychological mechanism of a high level of concern and to develop adaptive intervention strategies.

Most published work on the reproductive concerns of AYA cancer survivors consists of cross‐sectional studies. These studies provide further information on the prevalence of and factors associated with reproductive concerns in different areas of the world; in addition, these studies serve as a basis for population‐oriented interventions on reproductive concerns such as those with marriage, low education levels, desire to have biological children, breast cancer, poor family function, etc. However, there have only been four clinical intervention studies on this topic, which were all in China,^32^, ^42^, ^48^, ^70^ and the evidence is limited by the small participants of interventions and small sample sizes involved. Larger, adequately powered RCTs based on a theoretical framework are urgently required to develop effective interventions focusing on reproductive concerns and to decrease the negative effects of such high levels of concern on patients, families, and even society, hence, improving the quality of life of AYA cancer survivors.

Although fertility concerns have previously been partly reviewed within a systematic review of fertility‐related psychological distress,^15^ we felt we had to acknowledge the emerging nature of pertinent research. This scoping review conducted a systematic search of the literature specifically on the topic of reproductive concerns experienced by AYA cancer survivors, thereby collating the research to date. Although efforts were made in the search to include all relative studies, it is possible that some studies of reproductive concerns for AYA cancer patients have been missed. Our review included outcomes from quantitative, qualitative, and a few interventional studies, which enabled us to consider the breadth of previous study in an emerging field and to notify future researches by highlighting problems that should be understood when designing further studies in this field. On the other hand, this also means that the quality of the studies we surveyed has not been evaluated against the standards commonly used in systematic reviews.

Above all, the reproductive concerns of AYA cancer survivors are a psychological burden that urgently needs the attention of reproductive specialists, oncologists, psychologists, and nurses. But the limited number of studies to date have had small samples and were not designed to explore psychological processes involved or to construct a theoretical framework of reproductive concerns. Nonetheless, research with further clinical intervention trials needs to be conducted on cancer survivorship, centering on alleviating survivors' psychological distress or improving treatment outcomes. Further research into fertility consultations with AYA cancer patients in real clinical settings is also warranted.

## CONFLICT OF INTEREST

None.

## AUTHOR CONTRIBUTION

Sun and Xiao devised this study; Sun, Xie, Duan, Cheng, Luo, Zhou, and Liu searched the literature; Sun and Xie wrote the first draft of the paper; Xiao and Andy revised the paper. All authors reviewed the final paper.

## ETHICS STATEMENT

Neither informed consent to participate nor ethical approval is required.

## Supporting information


Table S1

## Data Availability

This review was based on published literature, all of which is fully listed.
