# Supplementary material for: Reproductive concerns among adolescent and young adult cancer survivors: A scoping review of current research situations
Source: Cancer Med. 2022 Mar 24;11(18):3508–17. doi: 10.1002/cam4.4708 (PMC9487873; doi:10.1002/cam4.4708)
Supplement: Supplementary file 1 — Table S1 [file CAM4-11-3508-s001.docx]

Table S1 Study Design, Measurement, and Outcomes of Studies Included in the Literature Review

| Publications | Country | Design | Type of Cancer and Age | Measurement(s) | Main Outcomes |
| --- | --- | --- | --- | --- | --- |
| Bártolo 2020 | Portugal | Cross-sectional | Female survivors of breast cancer, aged 18-40y (N = 43) | RCAC | Reproductive concerns fully mediated the relationship between the importance of parenthood in women’s lives and HRQoL |
| Bártolo 2020 | Portugal | Cross-sectional | Female survivors of breast cancer, aged 18-40 y (N = 104) | RCAC | Reproductive concerns fully mediated the relationship between the importance of parenthood in women’s lives and HRQoL |
| Bártolo 2021 | Portugal | Cross-sectional | Breast cancer survivors, aged 18-40 y (N = 38) | RCAC | The desire to have a (or another) biological child was a significant predictor of higher concerns related with fertility potential for all young women. Higher vigilance regarding reproduction-related cues seems to lead to higher concerns among women with breast cancer history whose fertility is threatened. |
| Benedict 2016 | United States | Cross-sectional | Male survivors of various cancers, aged 18-35 y (N = 346) | RCAC | Men who wanted to have a baby and men who believed that having a biological child was very important scored higher on at least one RCAC-Male subscale. We observed correlations in the expected directions between the reproductive concerns, depression and HRQoL. |
| Benedict 2016 | United States | Qualitative | Young adult survivors of adolescent cancers, aged 16-24 y (N = 43) | Semi-structured individual interviews and focus groups | Most AYAs in our study reported a number of reproductive concerns and fertility-related distress after cancer treatment, which may affect other areas of psychosocial functioning. Females may be more at-risk for distress than males, particularly in situations of uncertainty and limited knowledge. |
| Benedict 2018 | United States | Cross-sectional | Premenopausal females with a prior cancer diagnosis, aged 18-35 y (N = 346) | RCAC | Unaddressed fertility information needs, concerns, and decision distress may affect general QoL among post-treatment YAFCS who hope to have children in the future. |
| Burgmann 2018 | Germany | Cross-sectional | Female survivors of breast cancer, aged 23-46y (N = 160) | Self-designed scale | 76.4% of patients reported satisfaction with their decision. After BC diagnosis, 45.8% reported to have maternal desire, but only 21.7% actually planned to have children. 41.7% of patients missed sufficient counseling regarding parenthood concerns. |
| Carter 2010 | United States | Cross-sectional | Female cancer survivors (Gynecologic and Bone Marrow /Stem Cell Transplant), aged 18-49 y (N = 122) | RCS | No significant group differences were identified for mood, mental health QoL, reproductive concerns, and relationship satisfaction between cancer survivors and non-cancer infertile women. All groups scored in the FSFI range of sexual dysfunction, and with RCS scores above published means. |
| Carter 2007 | United States | Mixed method | Female survivors of cervical cancer, aged 23-40 y (N = 29) | FSFI, Semi-structured interviews | During the preoperative assessment, the majority of the women's concerns focused on their ability to conceive (67%, n=16) and maintain a pregnancy (58%, n=14). Concern about conception decreased after trachelectomy; however, as expected, concerns about pregnancy increased. Another reproductive issue concerned “time”, with subjects expressing a feeling of time pressure, i.e., “clock ticking”; “time limit within which to conceive”; and age. Others emphasized different time factors felt to be necessary prior to attempted conception, such as “worried that I may be too old by the time I get married” or “finding a partner”. Several women reported concerns about the impact of their cancer history or surgical treatment, i.e., “fear that something could go wrong with the baby as a result of the cancer or treatment”; “worry about the cerclage”, as well as concern about conception and recurrence. Regardless of these preoperative assessment misgivings, 85% (n=23) of the women provided a relatively high rating for future success of conception and childbirth (50-100%). |
| Chen 2020 | China | Cross-sectional | Female survivors of lung cancer, aged 18-45 y (N = 239) | RCAC | The young married female patients with lung cancer have a middle to high level of reproductive concerns and the total score of RCAC was 58.29±9.84, and the number of children and social relational quality entered the equation and explained 30.5% of the total variance. |
| Connell 2006 | Australia | Qualitative | Female survivors of breast cancer, aged ≤ 40 y (N = 13) | Semi-structured interviews | Over time not only did changes occur in fertility status of various participants but also in their perceptions of infertility as an issue. For some participants, infertility concerns remained constant, while other participants, who had in earlier interviews stated infertility was not an issue for them, had a change in perspective by the last interview. Such changes were centered around regret that fertility-preserving choices in early diagnosis had not been utilized. |
| Corney 2014 | United Kingdom | Qualitative | Female single childless survivors of breast cancer, aged 30-44 y (N = 10) | Semi-structured interviews | Partnership worries included concerns about having to undergo treatment without a partner to support them; the fear of rejection by potential partners; and feelings about the precious time lost in diagnosis and treatment. Fertility concerns included dilemmas about having children and feelings about the options of pursuing assisted reproductive techniques. |
| Crawshaw 2010 | United Kingdom | Qualitative | Young cancer survivors of various cancers, aged ≤30 y (N = 38) | In-depth single interviews | Four key experiences of managing fertility matters influenced, or were influenced by, the aftermath of cancer treatment: (1) prioritizing ‘normality’ and marginalizing fertility; (2) fertility concerns compromising ‘normality’; (3) ongoing impairments/health concerns mediating fertility matters; (4) fertility concerns dominating the cancer legacy. |
| Drizin 2021 | United States | Cross-sectional | Male survivors of various cancers, ages 18-35 y (N = 170) | RCAC | Young adult male cancer survivors who received FC were more likely to have high reproductive concerns than those who did not receive FC. Men who seek out FC after cancer diagnosis may need additional support for their reproductive concerns. |
| Ellis 2016 | Australia | Qualitative | Young cancer survivors of any cancer type, aged 12-20 y (N = 19) | Semi-structured interviews | Themes included: distress regarding potential infertility, the effect of infertility on future relationships and self-esteem; and miscommunications/confusion about fertility status, access to fertility testing and preservation options. |
| Ernst 2020 | Germany | Cross-sectional | Young cancer survivors of various cancers, aged 24-49 y (N = 632) | Cancer-specific version of the Leipzig Questionnaire of Motives to have a Child (LKM‐C) | Childhood cancer survivors reported more motives in favor of a child than worries. Favorable attitudes were associated with the presence of a partnership and children, stronger current desire for a child, and fewer depressive symptoms. Worries were associated with an unfulfilled desire to have a child and elevated levels of depression and anxiety symptoms. |
| Gorman 2010 | United States | Cross-sectional | Female survivors of breast cancer, aged ≤ 40 y (N = 131) | RCS | Greater recalled reproductive concerns as an independent predictor of consistent depressive symptoms after controlling for both social support and physical health. In bivariate analyses, being nulliparous at diagnosis and reporting treatment-related ovarian damage were both strongly associated with higher reproductive concerns and with depressive symptoms. |
| Gorman 2015 | United States | Cross-sectional | Female cancer survivors of various cancers, aged 18-35 y (N = 200) | RCAC | Of those with moderate to severe depression, 23% had high RCAC scores as compared to 6% of those with minimal to mild depression. A higher level of reproductive concerns was associated with greater odds of experiencing moderate to severe depression. |
| Gorman 2011 | United States | Qualitative | Female survivors of breast cancer, aged 26-38 y (N = 20) | Open-ended telephone interviews | The main themes were: 1) I was young, I wanted to do everything possible to move forward with my life and not to have the cancer come back; 2) Fertility concerns are different for every woman; 3) My oncologist was great… a huge part of my survivorship, and 4) They didn’t tell me about my options and I didn’t think about fertility until it was too late. |
| Guo 2019 | China | Cross-sectional | Female survivors of breast cancer, aged 21-40 y (N = 264) | RCAC | The scores of reproductive concerns, attachment avoidance and attachment anxiety were 57.49±6.11, 3.31±0.74 and 3.54±0.85, respectively. 105 participants showed safe attachment (39.8%). The multiple linear regression results revealed that fertility intention, attachment anxiety, number of chemotherapy and religion influenced the reproductive concerns in young breast cancer patients. |
| Hammond 2007 | United States | Prospective case-control cohort study | Cancer survivors who underwent myeloablative stem cell transplant, aged ≤ 40 y (N = 120) | Digital scoring method | 25% of survivors had moderate to high levels of concern about infertility, compared with 7% of controls. 54% of survivors younger than age 40 years expressed elevated infertility concern. Survivors without children before transplant had greater risk of elevated concern after 10 years. Although female controls were more likely to express elevated infertility concerns, there was no difference of concerns between male and female survivors. |
| Jardim 2020 | Brazil | Qualitative | Survivors of hematological malignancies, aged 18-24 y (N = 24) | Semi-structured interviews | Four themes were identified from the data: (1) knowledge about fertility; (2) emotional impact and fertility-related uncertainty; (3) sharing the possible risk of infertility with partners, and (4) need for information on possible loss of fertility. |
| Kang 2021 | China | Quasi-randomized control | Female survivors of breast cancer, aged 18-40 y (N = 106) | RCAC | The intervention group had higher Quality of Relationship Index score and lower Reproductive Concerns After Cancer scale score compared with the control group. |
| Kang 2021 | China | Cross-sectional | Female survivors of breast cancer, aged 21-40 y (N = 216) | RCAC | The score of reproductive concerns in young women with breast cancer at convalescence stage was (63.65±6.67), and the score of life quality was (104.30±10.56). And reproductive concerns was negatively correlated with QoL. |
| Kong 2021 | China | Cross-sectional | Female survivors of thyroid cancer, aged 18-30 y (N = 127) | RCAC | The total score of 127 young female thyroid cancer patients with fertility concerns was 64.59±9.08. The education level, number of children, and family function were negatively correlated with the level of fertility concerns in young women with thyroid cancer, while the degree of depression, whether radioactive I^131^ therapy was given or not was positively correlated with the level of fertility concerns. |
| Korte 2019 | Germany | Cross-sectional | Cancer survivors of any cancer type, aged 13-20 y (N = 142) | Self-designed scale | Both patients (86.1%) and parents (96.3%) indicated a strong desire for biological parenthood for themselves/their children. Patients who estimated their risk for fertility impairment being high were more likely to be concerned about their fertility. Parents who received fertility preservation information were more likely to recommend its use to their children, whereas parents of female patients were less likely to do so. |
| Li 2020 | China | Cross-sectional | Female survivors of various cancers, aged 18-45 y (N = 164) | RCAC | The scores of reproductive concerns and self-disclosure were 49.00 (42.00,57.75) and 38.04 (35.04,39.96), respectively; there was a negative correlation between reproductive anxiety and self-disclosure. There were significant differences in the scores of childbearing concerns among young female cancer patients with the same number of children, childbearing willingness and primary caregivers. |
| Ljungman 2018 | Sweden | Cross-sectional | Female survivors of breast cancer, aged 23-42 y (N = 181) | RCAC | A high level of reproductive concerns in at least one dimension was reported by 58%. Model results showed that current endocrine treatment was a significant predictor of dysfunction related to lubrication and vaginal discomfort. Negative body image was related to satisfaction with sex life. A high level of reproductive concerns was predicted by a wish for (additional) children in the future and by previous chemotherapy. |
| Ljungman 2019 | Sweden | Cross-sectional | Male survivors of testicular cancer, aged 18-42 y (N = 111) | RCAC | A high level of reproductive concerns was reported by 28%. Negative body image was associated with reproductive concerns in the dimensions of fertility potential, partner disclosure, and child’s health, whereas having had fertility preservation predicted higher levels of concerns with regard to personal health and achieving pregnancy. Clinical variables did not predict either sexual function or reproductive concerns. |
| Nilsson 2014 | Sweden | Qualitative | Survivors of various cancers, aged 16-24 y (N= 134) | Focus group discussions | The analysis resulted in the main category Is it possible to have a baby? including five generic categories: risk of infertility affects well-being, dealing with possible infertility, disclosure of possible infertility is a challenge, Issues related to heredity and parenthood may be affected. The risk of infertility was described as having a negative impact on well-being and intimate relationships. |
| Partridge 2004 | United States | Cross-sectional | Young women survivors of breast cancer with mean age of 32.9 y (N =657) | Self-designed scale | 57% recalled substantial concern at diagnosis about becoming infertile with treatment. Greater concerns about infertility was associated with wish for children or more children, number of prior pregnancies, and prior difficulty conceiving. Twenty-nine percent of women reported that infertility concerns influenced treatment decisions. 72% of women reported discussing fertility concerns with their doctors and 51% felt their concerns were addressed adequately. |
| Patterson 2020 | Australia | Cross-sectional | Cancer survivors of any cancer type, aged 15-29 y (N = 178) | Fertility Problem Inventory | Social concerns and expectations surrounding infertility have a negative impact on AYA cancer survivors' and patients’ QoL. |
| Qiao 2017 | China | Cross-sectional | Female survivors of various cancers, aged 18–40 y (N = 380) | RCAC | Young female cancer patients have higher reproductive concerns, with the overall reproductive concerns score for young female cancer patients was 57.26±8.34. Educational level, the number of children，fertility desire and types of cancer entered the multiple regression equations and explained the variance of 35.0%. Compared to breast cancer patients, thyroid cancer patients with lower level of reproductive concerns, while reproductive concerns scores of colorectal cancer and gynecological cancer patients showed no difference. |
| Qiao 2019 | China | Cross-sectional | Female survivors of various cancers, aged 18-40 y (N = 397) | RCAC | Young women with cancer have a higher degree of reproductive concerns was risk factors for depression. |
| Raghunathan 2018 | United States | Cross-sectional | Female survivors of various cancers, aged 18-35 y (N = 187) | RCAC | 65% reported concern about passing on a genetic cancer risk to their children, and scores did not vary regardless of association with cancer at high risk for genetic transmission. |
| Ruddy 2011 | United States | Cross-sectional | Female survivors of breast cancer, aged 31-43 y (N = 20) | Fertility Issues Survey | Survivors expressed greater concern about fertility compared with controls. Among survivors, 80% expressed some level of concern regarding fertility, compared with 25% of the controls. 16 survivors and 10 controls desired a future child. The desire to have a child in the future was associated with greater fertility concern in both survivors and controls, although this trend was statistically significant only in survivors. Of the 16 survivors who expressed concerns about fertility, 15 reported that they wanted a future child (or were unsure) and 1 reported that she did not (P=0.01). Of the 5 controls with fertility concerns, 4 reported that they wanted a future child and one reported that she did not (P=0.30). Among the women in both groups who desired a future child, survivors were more likely to express concerns about fertility. |
| Ruddy 2014 | United States | Prospective cohort study | Female survivors of breast cancer, aged 17-40 y (N = 724) | Fertility Issues and Outcomes Scale | Many young women with newly diagnosed breast cancer have concerns about fertility, and for some, these substantially affect their treatment decisions. Only a minority of female currently pursue available fertility preservation strategies in this setting. |
| Ruggeri 2019 | Switzerland | Cross-sectional | Female survivors of breast cancer, aged ≤ 40 y (N = 297) | Fertility Issues Survey | 67% discussed fertility issues before starting therapy, 64% were concerned about becoming infertile after treatment, and 15% decided not to follow prescribed therapies. 54% wished future children before diagnosis; of these, 71% still desired biologic children afterwards. In multivariable analysis, not having children was the only patient characteristic significantly associated with fertility concerns at diagnosis. 27% used fertility preservation strategies. Women who received chemotherapy reported greater physical and sexual difficulties than women who did not. Women who were married or had a partner reported less psychosocial problems than single women. |
| Russell 2016 | United States | Qualitative | Survivors of various cancers, aged ≥ 22 y (N = 56) | Semi-structured interviews | Lesbian, gay, bisexual, transgender, or queer cancer survivors’ views on relationships, parent-hood, and family building seemed to result in less distress when faced with infertility |
| Sella 2021 | United States | Prospective cohort study | Female survivors of breast cancer, aged 17-40 y (N = 643) | Self-designed scale | One-third indicated that fertility concerns affected endocrine therapy decisions. In multivariable analysis, only parity at diagnosis was significantly associated with fertility concerns affecting endocrine therapy decisions. Non-initiation/non-persistence was higher among women with fertility concerns versus those without. |
| Sobota 2021 | United Kingdom | Qualitative | Female survivors of gynecological or breast cancer, aged 18-45 y (N=24) | Semi-structured interviews | Five main themes pertaining to treatment-related decision-making experiences and fertility issues and fear of progression and recurrence: Becoming aware of infertility as a potential consequence of cancer treatment; Balancing-prioritizing cancer and fertility; Decisions about treatments; Evaluation of treatment decisions; and consequences of treatments. |
| Schover 1999 | United States | Cross-sectional | Survivors of various cancers, aged 18-45 y (N = 283) | Self-designed scale | Before cancer, 35% had at least 1 child, compared with 46% currently. Of those currently childless, 76% want children in the future. 19% have significant anxiety that their cancer treatment could impact negatively on their children’s future health. Of women, 18% fear that a pregnancy could trigger a cancer recurrence. Only 57% received information from their health care providers about infertility after cancer. Other reproductive concerns were discussed less often. Only 24% of childless men banked sperm before treatment. About 80% of the sample viewed themselves positively as actual or potential parents. Feeling healthy enough to be a good parent after cancer was the strongest predictor of emotional well-being as measured by the Mental Component Score of the SF-36. |
| Shah 2016 | United States | Prospective cohort study | Female survivors of non-gynecologic cancers, aged 18-40 y (N = 356) | RCS | Factors independently associated with higher RCS scores included a desire for children at the time of diagnosis, post-treatment infertility, treatment with chemoradiation or bone marrow transplant, and income <$100,000/year at diagnosis. Among the highest reported reproductive concerns were those related to loss of control over one’s reproductive future and concerns about effect of illness on one’s future fertility. Across our population and independent of age, in-depth reproductive health counseling prior to cancer treatment was associated with significantly lower RCS scores. Our findings highlight the importance of early counseling, and targeting high-risk groups for additional counseling after completion of cancer treatment. |
| Tang 2020 | China | Qualitative | Female survivors of thyroid cancer, aged 18-39 y (N = 19) | Semi-structured interviews | Young female patients with thyroid cancer have reproductive concerns and need for fertility knowledge generally, and three themes were extracted including worry and remorse, desire for communication and support, needs for scientific reproductive knowledge. |
| Villarreal-Garza 2017 | Mexico | Cross-sectional | Female survivors of breast cancer, aged 19-40 y (N = 134) | Fertility Issues Survey | At diagnosis, 44% of women expressed some level of concern about infertility risk. The only factor significantly associated with fertility concern was the desire of having children prior to diagnosis. Only 30.6% patients recalled having received information regarding infertility risk from their physicians. |
| Wang 2020 | China | Randomized Control Trial | Female survivors of gynecological malignant cancer, aged 18-40 y (N = 50) | RCAC | FC and education intervention can improve the level of cancer related fertility knowledge in young gynecological malignant cancer patients, and alleviate their reproductive concerns. |
| Wang 2019 | China | Quasi-randomized control | Female survivors of various cancers, aged 18-40 y (N = 72) | RCAC | Mindfulness-based stress reduction can effectively relieve reproductive concerns and fear of cancer recurrence for young female cancer patients. There were significant differences in spouse awareness，acceptance，pregnancy ability，own health，children health and total reproductive concerns scores between the two groups. The time effect on reproductive concerns of the various dimensions scores and total reproductive concerns scores was significant. |
| Wang 2020 | China | Cross-sectional | Female survivors of cervical cancer, aged 20-45 y (N = 390) | RCAC | The average reproductive concerns score of 285 cervical cancer patients of childbearing age was 56.45±8.18. Educational level, pathological type, clinical stage, surgical approach, the number of children and fertility desire entered the multiple regression equation and explained 38.0% of the total variance. |
| Wang 2021 | China | Qualitative | Female survivors of breast cancer, aged 24-40 y (N = 12) | In-depth single interviews | Four main themes pertaining to reproductive concerns: (1) Worry about yourself; (2) Concerns about children's health and care; (3) Fertility information support; (4) Negative emotional experience caused by infertility. |
| Wu 2019 | China | Cross-sectional | Female survivors of thyroid cancer, aged 18-40 y (N = 212) | RCAC | Young female patients with thyroid cancer had relatively high reproductive concerns with the total score of RCAC was (65.73±12.36), and education levels, number of children, childbearing intentions, use of I^131^ treatment and depression levels were contributing factors of reproductive concerns. |
| Young 2019 | China | Cross-sectional | Female survivors of various cancers, aged 18-40 y (N = 747) | RCAC | FC was reported by 19% of survivors and moderate to high overall reproductive concerns were reported by 44% of participants. Meanwhile, FC was significantly associated with moderate to high reproductive concerns. |
| Yuan 2018 | China | Cross-sectional | Female survivors of gynecological malignancy, aged 23-48 y (N = 256) | RCAC | The total score of reproductive concerns of female cancer patients was (56.58±6.52). The number of children, balanced families, intermediate families, gynecological malignancies and reproductive intention could affect the level of reproductive concerns of female cancer patients of childbearing age. |
| Zhang 2018 | China | Cross-sectional | Female survivors of various cancers, aged 22-46 y (N = 243) | RCAC | Young female cancer patients have higher reproductive concerns, with the overall reproductive concerns score of 62.83±5.78. The main influencing factors are work ability, family situation, sexual ability, communication ability and psychological condition. |
| Zhang 2020 | China | Randomized Control Trial | Female survivors of breast cancer, aged 22-40 y (N = 76) | RCAC | Mindfulness training can help improve breast cancer patients’ reproductive concerns and ruminate meditation, and enhance their hope index. |

RCAC, Reproductive Concerns After Cancer; RCS, Reproductive Concerns Scale; HRQoL, health-related quality of life; YAFCS, young adults female cancer survivors; AYA, adolescent and young adults; FSFI, Female Sexual Function Inventory; FC, Fertility counseling; SF-36, 36-Item Short Form Health Survey;
